# Relation between Perceived Barrier Profiles, Physical Literacy, Motivation and Physical Activity Behaviors among Parents with a Young Child

**DOI:** 10.3390/ijerph17124459

**Published:** 2020-06-21

**Authors:** Amy S. Ha, Wai Chan, Johan Y. Y. Ng

**Affiliations:** 1 Department of Sports Science and Physical Education, The Chinese University of Hong Kong, Shatin, Hong Kong; 2 Faculty of Education, The Chinese University of Hong Kong, Shatin, Hong Kong; waicwm@gmail.com (W.C.); yyng@cuhk.edu.hk (J.Y.Y.N.)

**Keywords:** physical activity barriers, parents, latent profile analysis, physical literacy, autonomous motivation

## Abstract

*Objectives*: to reveal distinct subgroups of parents by their perception of 6 types of physical activity barriers and challenges (i.e., lack of time, poor health, lack of company, lack of facilities, childcare responsibility, lack of motivation), and examine its relation with related constructs. *Design*: cross-sectional survey data. *Method*: the sample consisted of 424 parents who had at least 1 child of primary school age. Latent profile analysis was conducted to identify latent subgroups within participants. Group differences on physical literacy, autonomous motivation, and self-report physical activity (PA) levels were explored. *Results*: a four-profile solution was obtained from latent profile analysis, labelled as: “Struggling” (29.0%), “Family burden” (41.3%), “Lazy” (13.0%), and “Barriers free” (16.7%). The “Barriers free” profile experienced the least difficulties with physical activity, but the “Struggling” profile suffered the most severe barriers and challenges. “Family burden” and “Lazy” profiles demonstrated qualitative differences on one or two given challenges. Significant group differences on physical literacy, autonomous motivation, and PA levels were found, showing the “Barriers free” profile as the most robust and adaptive group of parents. *Conclusions*: the findings suggest that it is common for a substantial portion of parents to experience multiple barriers and challenges to a relatively high degree. Intervention on raising parent’s physical literacy to reduce barriers and sustain their motivation may be a target for intervention.

## 1. Introduction

### 1.1. Relation between Perceived Barrier Profiles, Physical Literacy, Motivation and Physical Activity (PA) Behaviors

Regular engagement of physical activity (PA) is imperative for one’s health, yet to many individuals, it is not without difficulties. Recent statistics showed that approximately a quarter of adults did not meet guidelines for PA, i.e., 150 min of moderate activity or 75 min of vigorous activity per week [1], even though the benefits of PA have been extensively documented and publicized [2,3,4]. These adults may struggle with challenges or barriers to PA so that they are not able to meet the recommendations. Indeed, barriers to PA undermine regular PA engagement but encourage a sedentary lifestyle, which may doubly jeopardize one’s health and mental wellness [5]. Barrier reduction programs to help adults overcome, or at least reduce, perceived barriers may differ based on the challenges they are facing (e.g., lack of skills versus lack of time). Therefore, identifying individuals facing similar challenges may be needed before group-specific interventions can be designed.

### 1.2. Barriers and Challenges to Physical Activities

A line of research inquiry has been exploring what and how challenges to PA affected whom across different settings, including both general populations stratified by sociodemographic differences (e.g., age, socioeconomic status, race/ethnicity) [6,7] as well as samples with specific developmental issues or clinical conditions (e.g., sedentary adolescent girls or older adults, people with serious mental illness or low income, stroke survivors, pregnant women, patients with osteoarthritis, African American women) [6,8,9,10,11,12,13,14].

Lack of time and motivation, and limited accessibility to facilities were the widely cited barriers and challenges to PA in adult groups [14,15,16]. Lack of energy, pain or symptoms related to a health issue, time constraints, and a lack of social support were typical PA challenges of clinical samples [8,9,12]. Individuals may experience different PA challenges specific to their developmental needs. Since one’s developmental stage or social role would define the context of PA barriers, it would be justifiable for researchers to explore PA challenges across diverse groups and hence to identify a subgroup of individuals who may often be challenged by any given PA barriers, in order to enhance the success of a lifestyle intervention program specific to the needs of the subgroup.

Apart from the aforementioned study samples, parents of young children have been gaining attention in the field when PA barriers are concerned. This is because most parents are often at risk for insufficient PA to achieve maximum health benefits, probably due to their concurrent occupational commitment and time deprivation for caregiving responsibilities [17,18,19]. Being a parent was identified as a risk factor for physical inactivity, with a small to moderate effect size obtained (summary *d* = 0.41) [17]. Even worse, parenthood may incur with sedentary lifestyles and physical inactivity. Compared to parents without a child in the household, parents living with a child together were two times more likely than others to sit and to have screen time for longer hours, but they were approximately 30% less likely to sleep for 7–9 h each night and to achieve an optimal level of PA [20]. As one of the most typical social roles, parents may suffer a great deal of PA barriers and challenges which may result in physical inactivity and hence undermine their health and quality of life over time.

Similar to prior studies, various PA challenges of parents are typical. These challenges included lack of time and social support, and fatigue [17]. Mailey et al. [19] identified multiple PA barriers from a group of working parents based on a qualitative study, including, family responsibilities, guilt (for making time for PA at the expense of work and family), lack of support, scheduling constraints, and work demands. Another study converged on the notion that a majority of parents (approximately 40–60%) reported that being busy with other responsibilities, lack of time, and fatigue were often perceived as barriers to PA [21]. Interestingly, these aforementioned PA barriers perceived by parents were also equivalent to those challenges of physical co-activity between parents and their children, or of PA of children themselves, namely facilities accessibility [22], and busy work schedules [23,24]. This lends some empirical support to the notion that PA challenges may be shared in the family. Lack of time emerged as one of the major challenges for parental PA. Lack of facilities was considered as another barrier that could prevent both parents and children from PA engagement together, especially when the built environment or weather failed to accommodate [5,25,26,27]. Thus, one would assume PA barriers perceived by parents may also adversely affect multiple family members, especially their children.

### 1.3. Potential Correlates of Physical Activity Barriers and Other Challenges

Physical literacy has been gaining attention recently in the field of sport and exercise science given its explanatory power to endeavors related to PA and sport, and even to health outcomes [28]. It was defined as a multifactorial construct with “the motivation, confidence, physical competence, knowledge and understanding to maintain physical activity throughout the life course” [29], consisting of physical, affective, and cognitive domains for a quantitative assessment [30,31]. Although the sub-constructs (e.g., motivation, competence) have been extensively studied in the field, the notion of studying the phenomenon as an umbrella construct related to physical activity has increased in recent years. Similar literacy constructs were well developed in other fields of literature. Nevertheless, multiple systematic reviews found that higher levels of physical literacy were positively associated with better PA and health outcomes [30,31].

In the adult population, past experiences could account for one’s physical literacy development, especially when favorable experiences in the past would facilitate physical literacy development [29]. Based on the conceptualization of physical literacy [32], knowledge and understanding, self-expression and communication with others, and sense of self and self-confidence are important subdomains. It is reasonable to assume those parents who were less physically literate or who had limited knowledge and understanding on PA, for example, were more likely to have limited resources (e.g., time, information) to cope with their PA barriers and hence to perceive those challenges to PA as more frequent or difficult. Since studies on both PA barriers and physical literacy together are lacking, the relation between PA barriers and physical literacy await further investigations.

Moreover, motivational regulation for PA may be another potential correlate to PA challenges. According to organismic integration theory [33,34], autonomous motivation, typically consisting of identified regulation (i.e., identifying the positive outcomes of PA) and intrinsic motivation (i.e., doing PA for the inherent enjoyment), is conducive to PA endeavors. To name but a few, multiple studies using objective, subjective measures, and systematic review analytic approaches converged on the notion that higher levels of autonomous motivation linked with greater PA levels in diverse groups such as adolescents [35] and parents [36,37,38]. By contrast, PA challenges were negatively related to both intrinsic and identified motivation among university students [39]. PA challenges were also inversely associated with leisure-time PA, but positively related to sedentary behaviors [5,16]. Since PA challenges would be a perception of reality to individuals, it is likely to undermine autonomous motivation in parents as well. Therefore, one would expect that PA challenges should be negatively related to autonomous motivation for PA. More empirical studies should be conducted to examine the linkage between PA challenges and autonomous motivation in parents.

Conceivably, PA challenges may also undermine the actual PA level. Being tired and having work demands were shown to undermine total PA, approximately having PA for more than 2.5 h per week, and these barriers increased the odds for sedentary behaviors [5]. Based on the accelerometry data, Müller and associates [40] demonstrated that more PA barriers in daily life (e.g., being tired, time constraints) were linked to shorter duration for weekly moderate to vigorous PA, even after statistically controlling for one’s motivation, suggesting that PA barriers could likely pose challenges in parents and lower their PA levels.

### 1.4. The Current Study: A Person-Centered Approach

A comprehensive examination of PA challenges perceived by parents is of great significance due to multiple considerations. First, pediatric obesity poses a public health concern in which parental obesity could double the risk for childhood obesity [41]. Health outcomes in children hinges on health of parents whose PA challenges are likely to affect adversely the PA levels of parents, in turn, to adversely affect their children. Therefore, PA challenges of parents may be a risk factor for pediatric obesity. Second, parents are often the gatekeeper or the agent for PA of young children, for example, their evaluation on facilities available for PA actually mattered more than that of children to both PA and health outcomes in children [22]. According to PA parenting perspective, parents could provide children with tangible support for PA and/or act as a role model [42,43,44,45], PA barriers could simply undermine the PA parenting role.

Third, PA challenges were demonstrated to be shared within the family. Wiseman and colleagues [46] showed that PA barriers of parents were associated with those of adolescents (Pearson correlation = 0.45, *p* < 0.01). In other words, PA barriers of parents could affect at least two generations. Children may be dependent upon some, if not all, PA endeavors of parents [47,48,49]. Reducing PA barriers in parents may be beneficial to both parents and their children [50]. Thus, it is essential for us to fully understand a parent’s barriers to PA before an intervention could be tailor made to improve the child’s PA outcomes via parental involvement. This study serves as an attempt to trace to the origin of physical inactivity in a family by uncovering PA barriers in parents.

A list of barriers and challenges to PA in parents were well documented in prior studies, to our best understanding, but very few studies directly and systematically shed light on these barriers to PA perceived by parents (e.g., lack of time, busy work schedule, lack of facilities). We are not entirely certain how many and the extent to which parents, in general, experienced different barriers to PA concurrently. Given that some parents may face greater levels of barriers and some others may not, it is reasonable to hypothesize for heterogeneity of parents in terms of severity of their barriers to PA (i.e., quantitative differences). We do not know whether or not any distinct subgroup of parents had fewer or more barriers to a varying degree, compared to other subgroups of parents (i.e., qualitative differences). To address these quantitative and qualitative issues enables a whole picture of challenges to PA perceived by parents and enriches our understanding of the proportion of parent population affected by different types of barrier to PA.

This present investigation adopts latent profile analysis (LPA) techniques to explore any distinct subgroups of parents due to their quantitative and qualitative differences in barriers to PA. This statistical analysis has been gaining popularity in the field of sport and health psychology [51,52,53]. A latent profile analytic approach is considered as robust and objective [54]. The main objective of the present investigation was to explore subgroups of parents with distinct barriers to PA profiles. Similar to other PA studies using LPA techniques [53], it was hypothesized that multiple groups would emerge based on types and frequency levels of challenges. At least a group with the highest level of challenges and a group with the lowest levels of challenges would be classified, we hypothesized that a relatively low proportion of parents would fall into either these two groups. One would also assume that there is another group with a medium level of PA barriers, and we hypothesized that a relatively high proportion of parents would belong to this profile. Then, a multinomial logistic regression was performed to explore what and how domains of physical literacy (i.e., knowledge and understanding, self-expression and communication with others, sense of self and self-confidence) predicted profile membership. In addition, multivariate analysis of variance (MANOVA) was conducted to explore any profile membership differences on PA motivation (i.e., identified vs. intrinsic), and behaviors. 

## 2. Methods

### 2.1. Participants

This study was based on data from a community intervention program to promote PA to primary students in school and family settings. The program began in Fall, 2017 and will run for 5 years. The intervention program involved 35 primary schools in Hong Kong. All parents of these primary students were invited to fill in an online questionnaire annually. The present set of analyses consisted of 424 parents, who responded between October 2018 and January 2019, and responded to all items of the assessment of parental PA barriers and challenges. These parents had at least one child at primary school age. The majority of respondents were mothers (81.4%), aged between 35–50 (81.1%), finished high school or above (98.3%), and stayed married (93.1%). Fewer than one half were employed full time (47.3%), and earned more than the median of household income (HK$35,500) of Hong Kong (44.3%) [55]. Ethical approval of the study was obtained from the authors’ university. Written informed consent was sought from respondents.

### 2.2. Measures

#### 2.2.1. Perceived Challenges to Physical Activity (6 Items)

A shortened version of the perceived barriers scale [56] was adapted to the current study. Six survey items were translated into Chinese and used to assess parent’s perceived difficulties on engaging PA. Parents were asked to report how often they were not able to engage in PA in the past week because of no time (“I haven’t got time”), poor health (“my health is not good enough”), no company (“there’s no one to do it with”), no facilities (“there’s no suitable facilities nearby”), childcare (“I’ve got young children to look after”), and lack of motivation (“I’m too lazy/not motivated/cannot get started) on a 5-point Likert scale (1 = never, 5 = everyday). The scale reliability was satisfactory (Cronbach α = 0.73).

#### 2.2.2. Physical Literacy (9 Items)

A scale of physical literacy was used to assess parent’s self-perception of fluency in PA knowledge and physical fitness [32]. Three domains, with 3 items each, measured knowledge and understanding (“I am aware of the benefits of PA related to health”), self-expression and communication with others (“I have strong social skills”), and sense of self and self-confidence (“I am physically fit, in accordance with my age”). Parents were asked to indicate the extent to which they agreed with each statement on a 5-point Likert scale (1 = *strongly disagree*, 5 = *strongly agree*). Mean scores were generated for these three domains. All three domains achieved satisfactory reliabilities (Cronbach’s αs ≥ 0.85).

#### 2.2.3. Autonomous Motivation (8 Items)

The intrinsic motivation (e.g., I exercise because it is fun) and identified regulation (e.g., I enjoy my exercise sessions) subscales of the Exercise Regulations Questionnaire [57] were applied to measure types of motivation for PA. Parents were asked to indicate the extent to which they agreed with each statement on a 5-point Likert scale (1 = *strongly disagree*, 5 = *strongly agree*). Both intrinsic and identified subscales reached acceptable reliabilities (Cronbach’s αs ≥ 0.63).

#### 2.2.4. Physical Activity Levels (7 Items)

Items adapted from the International Physical Activity Questionnaire (IPAQ) [58] were used to assess the intensity and duration for vigorous PA, moderate PA, and walking, in the past 7 days. A weighted total (vigorous = 8.0, moderate = 4.0, walking = 3.3) was used to estimate the metabolic equivalent of task (MET, minutes/week). A higher score represented higher PA levels.

### 2.3. Data Analysis

Ipsative standardization for scale items was performed before model estimation. These steps were taken for reducing response biases and for producing an easier interpretation [59]. Relevant survey items were subject to *z*-score transformation (*M* = 0, *SD* = 1). An individual mean and standard deviation were then calculated for each case. Each scale item was centered by individual mean and then divided by the dispersion.

Latent profile analysis techniques were applied to explore the optimal number of profiles based on those 6 perceived difficulties perceived by parents for engaging in PA. These profiles’ or subgroups’ membership is mutually exclusive with each participant who was assigned to one particular subgroup only. A given subgroup shared similarities on the study variables (i.e., PA barriers in the current study). This approach allows iterative processes to identify the best model with an optimal number of subgroup of respondents based on a number of fit indices for comparison with different models so that the model selection is subject more to fit criteria than exploratory purpose. Mplus version 8.2 was used. Solutions from 1 to 6 profiles were estimated. The estimation started with one profile, and one additional profile was added at a time to identify the best fitting model. Five fit indices were examined to determine model fit, i.e., Akaike’s information criterion (AIC), Bayesian information criterion (BIC), the sample size-adjusted BIC (SSA-BIC), boot-strapped likelihood ratio test (BLRT), and the Lo–Mendell–Rubin likelihood ratio test (LMR). Smaller values of AIC, BIC, and SSA-BIC demonstrated a model with better fit. BLRT or LMR enabled a comparison between models with or without an additional profile, and a non-significant test in BLRT or LMR indicated a model with an additional profile was not superior to that of without an additional profile, so the model with fewer profiles should be chosen. The entropy values (ranged from 0 to 1) were reviewed in order to examine the classification accuracy. Higher entropy values indicated greater accuracy of a given model. In addition to fit indices, profile parsimony and interpretation, and theoretical implications were taken into account to reach an optimal solution.

Multivariate tests were used to explore group differences of the resulting profiles on physical literacy as well as autonomous motivation as the potential correlates to PA barriers. Multinomial logistic regression was adopted to model physical literacy to predict profile membership using the profile with the highest level of PA barriers as the reference category. MANOVA or ANOVA techniques would be used to explore group differences on PA behaviors and autonomous motivation.

## 3. Results

Mean, standard deviations, and correlation matrices of all challenge items are summarized in the Table 1. For these descriptive analyses, raw scores were reported. On average, parents experienced all those challenges sometimes, but not more than very often. All forms of challenges were correlated (*r*s = 0.19–0.51, *p*s < 0.001), suggesting that those given challenges were somehow interrelated and that an underlying condition of parents might contribute to most challenges perceived by parents.

### 3.1. Latent Profile Analyses

**Figure 1:**
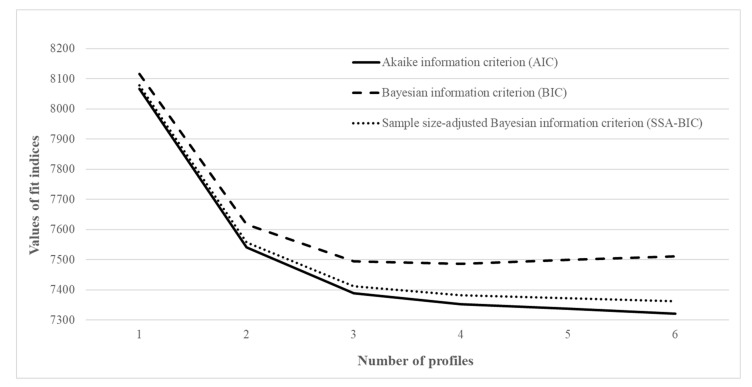
Elbow plot for the fit indices.

Profile solution results were summarized in Table 2. Almost all fit indices improved with an additional profile added. The entropy values were typical (≤0.751). However, LMR tests became non-significant with fifth profile added, suggesting the five-profile solution was not better than the fourth profile. Further profile divisions should be stopped. In a sense, adding up to fifth profile would result in two smaller profiles (approximately 10% or even smaller), which may dampen the class distinction and undermine the theoretical justification. Supplemental to the best solution identification, an elbow plot was portrayed in Figure 1, the drop of information criteria values leveled off from the third profile hereafter. Taken altogether, a four-profile model was chosen as the final model for further analyses.

**Figure 2:**
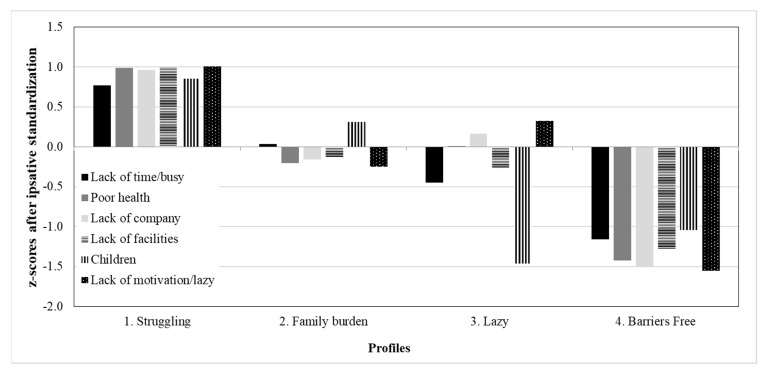
Characteristics of the latent profiles on the physical activity barriers perceived by parents.

The four-profile model was identified. Descriptive statistics of each profile of the final model are summarized in Table 3 and Figure 2. Since all variables were subject to *z*-score transformation and response bias reduction (i.e., ipsative standardization), the values above 0 (equivalent to the sample mean) represented positive values in standard deviation (SD) units, whereas the values below 0 represent negative values in *SD* units. Following the evaluation practices used by Gustafsson and colleagues [60], values from –5 to +0.5 *SD* were interpreted as slightly below or above the average, values within ±0.5 to ±1 *SD* were classified as low/high, values greater than ±1 *SD* were understood as very high. These interpretations provided readily comparisons between profiles by making a reference to the grand mean as the average standard of the sample.

Parents in profile 1 were considered as “Struggling” given that they reported on having 4 out of 6 barriers as very often (or at very high frequency), four of six challenges were very frequent to these parents (*SD*s = 0.79–1.07), being above average. Particularly, they experienced bad health, lack of company/facilities for PA, and low motivation. The remaining two barriers (i.e., busy work schedule, and babysitting burden) were also perceived as hindrances to PA with high frequency. Contrary to our hypothesis, the struggling profile had the second largest group membership across profiles.

Parents in profile 2 were labelled as having “Family burden”. They suffered from pressure related to childcare at a high frequency, slightly above the average. However, these parents suffered time constraints, no company/facilities, poor health or low motivation only on average or even slightly below average (*SD*s = 0.19–0.22). Consistent with our hypothesis, this profile had the largest group size.

Parents in profile 3 would be considered as being “Lazy”. The most distinguished feature for this profile would be their standing out challenge on having low motivation and a busy work schedule at a high frequency (*SD* = 0.45), while other challenges were just slightly above the average or even below the average (e.g., having no equipment/time for PA, and having (a) young child(ren) to take care at the same time; *SD*s = −1.66−0.21). This profile would serve as a big contrast to profile 2 “Family burden”.

Parents in profile 4 were labelled as “Barriers free”. Compared to the sample mean, these parents reported all six challenges which stopped them from doing PA at extremely low frequency, being very much below the average (*SD*s < −1.07). These parents may experience all challenges minimally.

Generally speaking, our research hypotheses were partially supported based on the latent profile analyses results. Multiple profiles were obtained. The “Struggling” profile represented parents who suffered all PA challenges at the worst level while the “Barriers free” profile represented those who suffered PA challenges the least. The “Family burden” and “Lazy” profiles suggested qualitative differences on PA challenges perceived by parents.

### 3.2. Group Differences on Correlates to PA Challenges

Multiple nominal regression techniques were used to model the profile membership with physical literacy as predictors. Results were summarized in Table 4. Indeed, parent’s challenges were well characterized by their levels of physical literacy. A one unit rise in the mean values in knowledge and understanding subdomain, was approximately one to four times more likely to be classified as other profiles than the “Struggling” profile. Similar patterns were also observed in other subdomains of physical literacy. To a certain extent, understanding and knowledge allowed the most consistent distinction between the “Struggling” profile versus all other profiles. However, the self-expression and communication with others failed to predict the relative membership likelihood into the “Lazy” and “Struggling” profiles, and that sense of self and self-confidence did not predict the membership of these two profiles characterized by medium to high levels of perceived barriers. These findings provided partial support for the inverse associations between PA challenges and physical literacy.

After identifying different groups of parents, we explored how motivation levels for PA would differ by profiles of the challenges perceived by parents. Group analyses using MANOVA were applied to examine two types of motivational regulations (i.e., identified vs. intrinsic). A significant overall group difference was detected: *F*(6, 838) = 9.26, *p* < 0.001. Post hoc analyses using Bonferroni adjustment demonstrated that the “Barriers free” profile displayed the highest level of autonomous motivation (i.e., identified, *F*(3, 420) = 4.19, *p* < 0.01; intrinsic, *F*(3, 420) = 18.88, *p* < 0.001; as seen in the Table 5). To note, the intrinsic type of motivation regulation demonstrated the greatest effect size on the comparison across profiles.

Furthermore, one way ANOVA results suggested group differences on PA levels (*F*(3, 420) = 4.84, *p* < 01), the post hoc comparison showed that the “Barriers free” profile were more physically active than the “Struggling” profile, but the differences on PA levels among the “Barriers free”, “Lazy”, and “Family burden” profiles were not statistically significant. Generally speaking, our research hypotheses were partially supported that autonomous motivation and PA levels on self-report were accounted for by PA barriers.

## 4. Discussion

The aim of the current study was to explore the distinct profiles of parents based on their self-report of six types of PA challenges, i.e., lack of time, poor health, lack of company, lack of facilities, the need for childcare, and lack of motivation. Then, their levels of physical literacy were compared across all profiles using multinomial logistic regression techniques. Multivariate techniques applied to explore any profile group differences on motivation for PA, and self-reported PA of these profiles. Partly consistent with the research hypotheses, four distinct profiles were identified. The “Struggling” profile represented a group of parents with all PA challenges at a very high frequency, while the “Barriers free” profile described a group of parents with all PA challenges at a relatively low frequency. The “Family burden” and “Lazy” profiles suggested two subgroups of parents who were qualitatively different from other profile groups, and who experienced a particular PA challenge occasionally. Generally speaking, compared to other profiles, parents in the “Barriers free” profile had greater levels of physical literacy, autonomous motivation, and PA than other three profile groups of parents. To our best understanding, this study served to be one of the first empirical studies characterizing multiple group profiles according to a combination of types and frequencies of common PA challenges, using a sample of parents. With the aid of latent profile modeling analyses, percentages of each profile were estimated which enabled our understanding on what and how these parents were affected by any given PA challenge(s). These profile classifications may pose significant theoretical and practical implications.

One novel finding from the current study was to provide estimates on the proportion of each profile. Partly consistent with our hypotheses, at least one group of parents were least affected by PA challenges (i.e., “Barriers free”), and a group of parents suffered the worst PA challenges (i.e., “Struggling”). The “Family burden” or “Lazy” profiles emerged in the middle in between two opposing profiles along the continuum, with lesser PA challenges than the “Struggling” profile, but not as adaptive as the “Barriers free” profile. Even so, the “Struggling” and “Barriers free” profiles shared a similarity that they were badly affected or less affected by all six challenges at the same time and all these profiles added up to more than 40% of the entire sample. One would query whether these parents may perceive different types of PA challenges as either equally difficult or negligible. Such perception may allude to an overall attitude of parent towards difficulty of regular PA engagement instead of experiences with those specific PA challenges, these claims should be verified with further investigations.

Evidently, the “Struggling” profile represented the most powerless parents with regard to PA engagement. They had a broad range of PA challenges to a relatively severe extent. It is uncertain which challenge posed the worst/least difficulty to these parents, which suggested constraints for a barrier reduction intervention program for these parents given their multiple pathways or mechanisms leading to PA barriers [21]. Nevertheless, future studies should shed a light on this particular profile group and understand their underlying needs in order to tackle their PA barriers.

To note, the largest group was the “Family burden” profile which contained more than 40% of the entire sample. These parents specified childcare as a main hindrance to their PA engagement. They might perceive taking care of a young child as a source of competition for their availability and energy for PA. They might also feel that they would have to make a choice between their family duties versus their own PA. In a sense, family duties and PA engagement may seem to be mutually exclusive to each other to these parents. Nevertheless, recent literature has been proposing family sport or workouts with family members [61]. A family-based intervention program with multi-component might be particularly meaningful to these parents. Such intervention design may fit the context of Hong Kong or other regions. A protocol study proposed that easy sport equipment, PA classes conducted in the school delivered by a coach cultivated in a train-the-trainer scheme, and provision of online PA materials for primary school children and their families in Hong Kong may alleviate the parental constraints for PA engagement [62]. Similar practices could be implemented in other feasibility studies.

More importantly, the current study sought to fill in the knowledge gap by addressing potential antecedents of these four profiles of parents, i.e., physical literacy. To be specific, knowledge and understanding of PA concepts appeared to have the greatest explanatory power for the profile patterns. Based on a recent meta-analytic study [30], very few studies measured physical literacy in adults, but PA level is a typical correlate to physical literacy, so the current findings await further verification from future studies. Nevertheless, one would assume parents who are more physically literate would be more able to cope with their challenges in reality by applying appropriate strategies (for example, integrating PA to the daily routines, better time management, focusing on some specific types of PA alone with minimal use of equipment if companions and facilities are concerns) in order to minimize the impacts of PA challenges so that it would be more likely for these parents to stay free of barriers. Conversely, parents who were less physically literate might perceive their barriers to PA as less surmountable, in turn, these challenges prevent them from engaging in PA regularly in reality. In a sense, improving one’s physical literacy may empower those parents who are struggling with PA challenges. Targeting one’s knowledge and understanding of PA may be a very first step to cope with challenges effectively.

In this study, we also made an attempt to explore group differences on autonomous motivation. As expected, the “Barriers free” profile demonstrated the highest level of both identified and intrinsic motivation, while the autonomous motivation of the “Struggling” profile were very much undermined. It is emphasized that the current study was merely a correlational study in nature, and these findings did not warrant causal relationships between PA challenges and the motivation regulation. Despite the cross-sectional design, it is rational to assume that these perceived PA challenges reflect the reality of these parents to a certain degree. They were not self-efficacious to cope with PA challenges so that they would perceive those challenges and PA engagement as overwhelming [21,63,64]. In a sense, the PA challenges, as a subjective reality, were detrimental to their motivation for further PA engagement. Such reasoning could be reversed by claiming parents who had high autonomous motivation were more determined to cope with PA challenges, in a sense that motivation could also act as a protective factor to PA challenges [34]. Regardless, the linkage between PA challenges and physical literacy may help to set forth a procedure to sustain parents’ PA, i.e., a mitigation of PA challenges may pave a foundation of developing one’s autonomous motivation for PA and subsequent PA engagement. At the same time, including prioritization and planning techniques as a chapter for a physical literacy curriculum may be feasible to raise PA levels in parents [21]. Future studies should seek to verify the temporal precedence between PA challenges and motivation regulations.

Another set of findings would be the group differences on PA levels. We are able to estimate the proportion of parents (i.e., the “Struggling” profile) who suffered the worst challenges and in turn whose PA was very much undermined compared to other profile groups. Those parents who were “Barriers free” were indeed more physically activity. This would urge the need for intervention for parents from the “Struggling” profile group as their challenges, did translate to their physical inactivity, which may pose implications for public health, especially among middle-aged adult with a primary school child. Future studies should seek to examine whether an intergenerational transmission of physical (in)activity to children was due to the parental PA challenges by including more child-related PA outcomes in analyses.

The present investigation contained several limitations. First, the study captured six PA challenges only, despite the fact that these six challenges were the most frequently cited from the extant literature. It is still possible for an individual to have many more different PA challenges than those listed. Future studies should expand the measurement scope of PA challenges and replicate whether the current latent profile classifications are still upheld when more PA challenges are considered. Then, all measures were self-reported, and it is inconclusive to decide whether the actual PA levels would vary across these group profiles of parents. The current study was also correlational in nature, and causal references on the relationships between physical literacy, PA challenges, and autonomous motivation could not be drawn. We were not able to explore whether and how the PA barriers and other challenges vary over time. A longitudinal study is needed for showing the temporal sequence of contribution of physical literacy to PA challenges which could subsequently dampen motivation. Moreover, some measures did not exhibit excellent psychometric properties specific to this parent sample (e.g., Cronbach’s alpha scored slightly below 0.7 for the autonomous motivation construct). Another scale with a higher proven reliability should be applied in future studies specific to the parent sample. Also, the current sample was limited to parents self-reporting from a small region in Asia. These parents might be relatively homogenous in terms of race or ethnicity background (e.g., Chinese/Cantonese speaking). Findings generalizability should be verified in other racial groups. One possible direction for future research is to examine the interplay between parents’ and their children’s perceptions. Indeed, children’s PA development hinges on parental PA development [23,25,44,49], so it is still worthwhile for researchers to investigate the PA development of parents first as in the present investigation. Nonetheless, the current study may serve as a stepping stone to explore how parental PA challenges determine children’s PA barriers. To fill in the puzzle, it would also be interesting to investigate how these group profiles of parents accounted for children’s PA values and their actual PA engagement.

## 5. Conclusions

In summary, parents suffered a varying degree of multiple PA challenges. This study adopted a person-centered approach to reveal distinct groups of parents by their types and frequency of PA challenges. The emergent profiles informed us of the proportion of what and how each profile of parents perceived their PA challenges. One important next step would be an exploration of connections of parents’ PA challenges to children’s PA endeavors in order to provide guidelines for relevant intervention programs for families with PA challenges as a whole.

## Figures and Tables

**Table 1:** Correlations matrices.

Variables	1	2	3	4	5	M	SD
1. Lack of time	-					3.38	0.91
2. Poor health	0.45 ***	-				2.98	0.75
3. Lack of company	0.26 ***	0.35 ***	-			3.00	1.00
4. Lack of facilities	0.22 ***	0.35 ***	0.46 ***	-		2.52	0.95
5. Children	0.39 ***	0.24 ***	0.24 ***	0.28 ***	-	3.61	1.24
6. Lack of motivation	0.25 ***	0.51 ***	0.41 ***	0.37 ***	0.19 ***	3.16	0.91

M = Mean; SD = Standard Deviation.0. ***p < 0.001.

**Table 2:** Latent profile fit statistics.

No. of Profile(s)	Free Parameters	LL	Scaling	AIC	BIC	SSA-BIC	Entropy	LMR	BLRT	<5%
1	12	−4021.317	0.9039	8066.633	8115.230	8077.150	-	-	-	-
2	19	−3751.203	1.2010	7540.407	7617.352	7557.058	0.764	0.000	0.000	0
3	26	−3667.783	1.1173	7389.566	7494.859	7412.352	0.777	0.001	0.000	0
**4**	**33**	**−3643.307**	**1.1534**	**7352.614**	**7486.255**	**7381.534**	**0.752**	**0.049**	**0.000**	**0**
5	40	−3628.445	1.5583	7336.890	7498.879	7371.945	0.755	0.838	0.000	0
6	47	−3613.211	1.2635	7320.421	7510.759	7361.611	0.751	0.196	0.000	0

LL = Log-likelihood; AIC = Akaike information criterion; BIC = Bayesian information criterion; SSA-BIC = sample size-adjusted Bayesian information criterion; LMR = Lo–Mendell–Rubin adjusted likelihood ratio test; BLRT = boot-strapped likelihood ratio test. Bold corresponds to the model with the best fit.

**Table 3:** Descriptive statistics on the 5 latent profile solution (*n* = 424).

Items ^1^	Struggling (*n* = 123; 29%)	Family Burden (*n* = 175; 41.3%)	Lazy (*n* = 55; 13%)	Barriers Free (*n* = 71; 16.7%)
1. Lack of time	0.79	0.02	−0.49	−1.15
2. Poor health	1.01	−0.20	0.08	−1.48
3. Lack of company	1.02	−0.19	0.21	−1.51
4. Lack of facilities	1.01	−0.11	−0.32	−1.28
5. Children	0.89	0.31	−1.66	−1.07
6. Lack of motivation	1.07	−0.29	0.45	−1.57

^1^ z-scores after ipsative standardization.

**Table 4:** Multinomial logistic regression on group membership by domains of physical literacy.

	Family Burden vs. Struggling				Lazy vs. Struggling				Barriers Free vs. Struggling			
	*B*	*SE*	OR	95% CI	*B*	*SE*	OR	95% CI	*B*	*SE*	OR	95% CI
Physical Literacy												
Knowledge and understanding	0.48 *	0.21	1.62	1.06–2.46	0.65 *	0.29	1.91	1.08–3.35	1.43 ***	0.33	4.17	2.20–7.90
Self expression and communication with others	0.38 *	0.19	1.46	1.00–2.13	0.39	0.26	1.47	0.90–2.43	0.60 *	0.27	1.82	1.08–3.09
Sense of self and self-confidence	0.79 ***	0.19	2.21	1.53–3.19	0.38	0.24	1.46	0.92–2.34	1.47 ***	0.27	4.34	2.57–7.33

***B*** = Unstandardized regression coefficient, ***SE =*** Standard Error, OR = Odds Ratio, 95% CI = 95% Confidence Interval; * *p* < 0.05, *** *p* < 0.001.

**Table 5:** Group differences on types of physical activity endeavours ^1^.

	Struggling (*n* = 123; 29.0%)		Family Bburden (*n* = 175; 41.3%)		Lazy (*n* = 55, 13.0%)		Barriers Free (*n* = 71; 16.7%)		*F*	Post Hoc	Partial η ^2^
	*M*	*SD*	*M*	*SD*	*M*	*SD*	*M*	*SD*			
Autonomous motivation									9.26 ***		
Identified	−0.13	0.53	0.02	0.57	−0.07	0.54	0.15	0.60	4.19 **	4 > 1	0.029
Intrinsic	−0.31	0.62	0.09	0.64	−0.04	0.67	0.33	0.51	18.88 ***	4 > 2 = 3 > 1	0.119
Total PA	98.85	62.35	118.35	65.39	109.94	69.11	137.72	93.21	4.84 **	4 > 1	0.033

^1^*F*-statistics were based on multivariate analysis of variance (MANOVA)/ANOVA. ^2^ Bonferroni adjustment was conducted for post hoc comparisons. Sample sizes varied slightly due to missingness. ***p < 0.001.
